# Comparative Studies on Bioactive Constituents in Hawk Tea Infusions with Different Maturity Degree and Their Antioxidant Activities

**DOI:** 10.1155/2014/838165

**Published:** 2014-07-15

**Authors:** Ming Yuan, Xuejing Jia, Chunbang Ding, Shu Yuan, Zhongwei Zhang, Yanger Chen

**Affiliations:** ^1^College of Life Sciences, Sichuan Agricultural University, Ya'an 625014, China; ^2^College of Resources and Environmental Science, Sichuan Agricultural University, Chengdu 611100, China

## Abstract

Hawk tea (*Litsea coreana* var. *lanuginose*) is a very popular herbal tea in the southwest of China. According to the maturity degree of raw materials, Hawk tea can usually be divided into three types: Hawk bud tea (HB), Hawk primary leaf tea (HP), and Hawk mature leaf tea (HM). In this study, some of the bioactive constituents and antioxidant properties of the three kinds of Hawk tea infusions were comparatively investigated. The results showed that the contents of total flavonoids, vitamin C, and carbohydrates in Hawk bud tea infusion (HBI) were higher than those in Hawk primary leaf tea infusion (HPI) and Hawk mature leaf tea infusion (HMI). HPI had higher contents of total polyphenols and exhibited better DPPH radical scavenging activity and ferric reducing activity power. HBI could provide more effective protection against erythrocyte hemolysis. As age is going from bud to mature leaf, the ability to inhibit the formation of low density lipoprotein (LDL) conjugated diene and the loss of tryptophan fluorescence decreased. The bioactive constituents and antioxidant activities of Hawk tea infusions were significantly affected by the maturity degree of the raw material.

## 1. Introduction

Generally, the production and scavenging of free radicals keep dynamic balance in human body. However, massive free radicals can be generated in cellular metabolic process under the stress condition [1]. And excessive free radicals can induce damages to cells or tissues and destroy the structure and function of biological macromolecules, which can cause various diseases [2]. Fortunately, the antioxidants can help cells keep the normal metabolic system in face of excessive free radicals. In recent years, herbal tea steps into the public eyes because of its high antioxidant activity and other health benefits [3]. There are a mass of flavones or other polyphenols in herbal tea. It was reported that phenolic compounds could reduce the risk of cardiovascular disease or cancer, which might be related to their antioxidant activity [4]. There are various methods to evaluate the antioxidant capacity in vitro. These methods can be classified as either hydrogen atom transfer (HAT) reaction or electron transfer (ET) reaction. They are widely accepted by many researchers and applied in immense amounts of researches [5].

Hawk tea is one of the most popular herbal teas in the southwest of China. Hawk tea is made from buds or leaves of* Litsea coreana* var.* lanuginose*, and this arbor is widely distributed in the mountainous area of south China [6]. Hawk tea infusion with a slight camphor-aromatic smell is free from caffeine and can reduce blood sugar or blood lipid [7]. Due to the different harvest time, Hawk tea can be classified into three types: Hawk bud tea (HB), Hawk primary leaf tea (HP), and Hawk mature leaf tea (HM). Their price fluctuates dramatically due to their yields. In the local markets, the price of HB, HP, and HM is about $50/kg, $20/kg, and $3/kg [8], respectively. 17 kinds of amino acids and plenty of beneficial mineral elements were found in HP [9], and 32 compounds were identified from the essential oil of HP [10]. However, these researches were all only using HP as the material, and the other two types of Hawk tea were overlooked entirely. As a popular traditional beverage, there are more than 30 million drinkers of this beverage [6]. Therefore, it is necessary to investigate the health benefits of Hawk tea infusions.

The objective of this work was to compare the bioactive constituents in three kinds of Hawk tea infusions systematically. In addition, their antioxidant properties (DPPH radical scavenging activity, ferric reducing activity power, and inhibition of erythrocyte hemolysis and LDL oxidation) were also investigated and the correlation between the constituents and antioxidant activity was discussed as well.

## 2. Materials and Methods

### 2.1. Reagents and Materials

Trolox (6-hydroxy-2,5,7,8-tetramethylchroman-2-carboxylic acid), DPPH (1,1-diphenyl-2-picrylhydrazyl), TPTZ (1,3,5-tri(2-pyridyl)-2,4,6-triazine), and AAPH (2,2′-azobis(2-methylpropionamidine) dihydrochloride) were purchased from Sigma (St. Louis, MO, USA). Ultrapure water was used throughout and obtained from a Milli-Q system.

Hawk teas (*Litsea coreana* var.* lanuginose*) were grown in Shangli town (N30°08′12.89′′, E103°04′16.57′′). The buds, primary leaves, and mature leaves of Hawk tea were harvested in around February, March, and May 2013, respectively. Processed Hawk teas under the same condition were all purchased from the same local retail shop (Yucheng District, Sichuan Province, China) in August 2013. Prior to sample preparation, the teas were ground into homogeneous and fine powder using a pestle and mortar and stored in a desiccator for further analysis.

### 2.2. Determination of Bioactive Constituents of Hawk Teas Infusions

Briefly, 0.5 g sample was blended with 50 mL deionized water at 90°C and gently agitated for 25 min. After cooling, infusions were centrifuged at 2500 r/min for 10 min, and then the supernatant was prepared for the following analysis and named Hawk bud tea infusion (HBI), Hawk primary leaf tea infusion (HPI), and Hawk mature leaf tea infusion (HMI), respectively.

*Determination of Protein.* Content of protein was analyzed according to the Bradford method [11] using bovine serum albumin (BSA) as the standard. Briefly, 10 *μ*L sample was added to 5 mL Coomassie Brilliant Blue G-250 solution (0.1 mg/mL) in a 10 mL cuvette. The mixtures were shaken vigorously and incubated in the dark for 5 min. The absorption was measured at 517 nm using the spectrophotometer (UV-1750, Shimadzu). Values were calculated according to the calibration curve (*y* = 0.0052*x* + 0.0047, *R* ^2^ = 0.996).

*Determination of Vitamin C.* Content of vitamin C was determined based upon the quantitative decolouration of 2,6-dichlorophenol indophenol [12] using ascorbic acid as the standard. Briefly, 0.5 mL sample was added to 0.5 mL 2,6-dichlorophenol indophenol dyeing solution (0.1 mg/mL) in a 10 mL cuvette and incubated for 5 min at room temperature. After that, 5 mL xylene was added. The mixtures were shaken vigorously for 1 min. The excess dye was extracted by xylene and the absorption was measured at 500 nm using the spectrophotometer (UV-1750, Shimadzu). Values were calculated according to the calibration curve (*y* = 0.0053*x* + 0.0165, *R* ^2^ = 0.992).

*Determination of Carbohydrates.* Content of carbohydrates was determined by phenol-sulfuric acid colorimetric method [7] using D-glucose as the standard. Briefly, 0.8 mL sample was added to 0.4 mL phenol solution (50 mg/mL) in a 10 mL cuvette and incubated for 5 min at room temperature. After that, 2 mL concentrated sulfuric acid was added. The mixtures were shaken vigorously for 1 min. The absorption was measured at 490 nm using the spectrophotometer (UV-1750, Shimadzu). Values were calculated according to the calibration curve (*y* = 0.004*x* + 0.001, *R* ^2^ = 0.997).

*Determination of Total Flavonoids.* Content of total flavonoids (TFC) was determined by the spectrophotometric method [13] using rutin as the standard. Briefly, 0.25 mL sample was added to 75 *μ*L NaNO_2_ (50 mg/mL) in a 5 mL cuvette. After that, 150 *μ*L Al (NO_3_)_3_ (100 mg/mL) was added and incubated for 5 min at room temperature. 0.5 mL NaOH (1 mol/L) and 2.5 mL deionized water were added and mixed. The absorption was measured at 510 nm using the spectrophotometer (UV-1750, Shimadzu). Values were calculated according to the calibration curve (*y* = 0.007*x* + 0.024, *R* ^2^ = 0.999).

*Determination of Total Polyphenols.* Content of total polyphenols (TPC) was determined by the spectrophotometric method [14] using gallic acid as the standard. Briefly, 50 *μ*L sample was added to 0.5 mL Folin-Ciocalteu solution (1 mol/L) in a 5 mL cuvette and incubated for 5 min at room temperature. After that, 1.5 mL NaNO_2_ (100 mg/mL) and 1 mL deionized water were added and mixed. The absorption was measured at 700 nm using the spectrophotometer (UV-1750, Shimadzu). Values were calculated according to the calibration curve (*y* = 0.007*x* − 0.006, *R* ^2^ = 0.998).

### 2.3. Determination of Antioxidant Capacity

*DPPH Radical Scavenging Activity.* DPPH radical scavenging activity was evaluated according to the previous report in [15]. Briefly, 1 mL sample solution was added to 3 mL DPPH methanol solution (0.05 mmol/L) in a 5 mL cuvette. The mixtures were shaken vigorously and incubated in the dark for 20 min. After that, the reduction of DPPH radical absorption was measured at 517 nm using the spectrophotometer (UV-1750, Shimadzu). The scavenging activity on DPPH radical was calculated by the following equation:

(1) DPPH  radical  scavenging  activity  (%) =  [ADPPH−AsampleADPPH]×100,

where *A*_DPPH_ is the absorbance of the DPPH radical solution without sample and *A*_Sample_ is the absorbance of the DPPH radical solution with tested samples.

*Ferric Reducing Activity Power Assay.* Ferric reducing activity power (FRAP) was evaluated according to the previous report in [16] using FeSO_4_·7H_2_O as the standard. The fresh FRAP reagent was prepared before using, which contains 25 mL acetate buffer (300 mmol/L, pH 3.6), 2.5 mL TPTZ solutions (10 mmol/L in 40 mmol/L HCl), and 2.5 mL of FeCl_3_ *·*6H_2_O solution (20 mmol/L). The reagent was warmed to 37°C, and then 500 *μ*L was placed in a cuvette and the initiate absorbance was read. 20 *μ*L of the sample solutions was added to the cuvette and the absorption was determined at 593 nm using the spectrophotometer (UV-1750, Shimadzu). Values were calculated according to the calibration curve (*y* = 0.6786*x* − 0.0062, *R* ^2^ = 0.997).

Erythrocyte hemolysis was based on the previous method in [17] using ascorbic acid as the positive control. Briefly, 200 mmol/L AAPH was added to 10% suspension of erythrocytes and then incubated for 2 h at 37°C. The absorbances *A* (0.15 mol/L NaCl) and *B* (deionized water) of the supernatant were read at 540 nm. The percentage hemolysis was calculated by the equation (1 − *A*/*B*) × 100%; ascorbic acid was used as a positive control.

Low density lipoprotein (LDL) oxidation was based on the previous method in [18] using Cu^2+^ as the positive control. To monitor conjugated diene, lag time was determined. Briefly, 0.01 mL 0.1 mmol/L Cu^2+^ (final concentration 5 *μ*mol/L) was added to the mixture that contained 0.02 mL LDL (100 *μ*g protein/mL) and 0.01 mL sample. To monitor tryptophan fluorescence, the residual tryptophan fluorescence was expressed. Briefly, 0.01 mL 0.1 mmol/L Cu^2+^ (final concentration 5 *μ*mol/L) was added to the mixes that contained 0.014 mL LDL (125 *μ*g protein/mL) and 0.02 mL sample.

### 2.4. Statistical Analysis

Experiments were carried out in triplicate, and data were reported as means ± standard deviation (SD) and evaluated by Student's *t*-test. The *P* values were set at *P* < 0.05 to assess the statistical significance.

## 3. Results and Discussion

### 3.1. Effects of Maturity Degree on Bioactive Constituents of Hawk Tea Infusions

As shown in Table 1, the bioactive constituents of Hawk tea infusions were affected dramatically by the maturity degree of the raw material (*P* < 0.05).

The protein contents ranged from 3.44 to 14.36 mg/g. The highest protein content was found in HBI (14.36 mg/g), while the lowest protein content was found in HPI (3.44 mg/g). The vitamin C contents of three kinds of Hawk tea infusions varied from 3.15 to 21.67 mg/g. The highest level of vitamin C was found in HBI, while the lowest level was in HMI. The highest carbohydrate content was found in HBI (82.79 mg/g), which was more than twice that in HMI (35.97 mg/g).

The results showed that tender leaves had more vitamin C contents than mature leaves. Commonly, the more mature the leaves, the higher the vitamin C content. When it comes to the infusion, it is the opposite. This phenomenon might be related to the available dissolution rate of vitamin C. Mature leaves had more cellulose and pectin which were the main components of cell wall. In addition, vitamin C mainly existed in plant cell wall [19]. Vitamin C in tender leaves might be easily released because of its less cellulose and/or pectin, and so were protein and carbohydrate. Another reason might be due to the difference of their water content in fresh material, and in general tender leaves contained much more water than mature leaves.

Polyphenols in tea primarily include flavonoids, phenolic acids, tannins, and lignans [20]. The level of total polyphenols fluctuated dramatically in Hawk tea infusions and ranged from 4.22 to 79.01 mg/g (Table 1). The highest level of total polyphenols was found in HPI, while the lowest level was in HMI. These results indicated a general trend that young material contained higher level of polyphenols.

Chiu and Lin [21] found that the content of total polyphenols in young leaf was much higher than that in old leaf of Tung-Ting tea. Total polyphenols of the fermented tea whose raw material came from dry season were significantly higher than those from monsoon season [22]. Therefore, the content of total polyphenols may be affected by species, region, climate, and maturity degree. In our results, young leaf infusion (HPI) showed a higher level of total polyphenols than the mature leaf infusion (HMI), but the more tender material infusion (HBI) contained less polyphenols than young leaf infusion (HPI). Since the bud is composed of unexpanded leaves and undeveloped stem, the undeveloped stem may have much less polyphenols than leaf. Therefore, although HB was younger than HP, HBI showed a lower level of polyphenols than HPI.

Flavonoids are the main groups of polyphenols and they are important antioxidants due to their high redox potential [23]. The main groups of flavonoids in Hawk tea are quercetin, quercetin-3-o-*β*-D-galactopyranoside, kaempferol-3-o-*β*-D-glucopyranoside, quercetin-3-o-*β*-D-glucopyranoside, and kaempferol-3-o-*β*-D-galactopyranoside [24]. Yu and Gu [9] found that the content of total flavones in Hawk tea was about triple that in green tea. HBI was the richest source of total flavonoids, 24.65 mg/g, while HMI was the lowest, just 1.25 mg/g (Table 1). The ratio of total flavones to total polyphenols in HBI was very high, which accounted for almost half of the total polyphenols. The similar trend was also found that young raw material contained more flavonoids than the aged raw material.

### 3.2. Effects of Maturity Degree on Antioxidant Activities of Hawk Tea Infusions

*DPPH Radical Scavenging Activity*. The results were shown in Table 2; the scavenging effects of Hawk tea infusions were well correlated with the concentration up to 0.5 mg/mL. HPI had the strongest activity, while HMI was the lowest (*P* < 0.05). At 0.5 mg/mL, the scavenging activities of HMI, HPI, and HBI were 75.7%, 92.0%, and 86.2%, respectively. So HMI was the weakest electron donor among them. Older raw material showed weaker DPPH free radical scavenging activity. These results are in line with the trend of contents of polyphenols, so DPPH free radical scavenging activity may be closely related to the polyphenols in Hawk tea.

*FRAP Values*.  Table 3 showed that the reducing power of Hawk tea infusions was positively correlated with the increased concentration up to 0.5 mg/mL. The FRAP values of HPI, HBI, and HMI were 1.9 mmol/L, 2.6 mmol/L, and 1.7 mmol/L at the concentration of 0.5 mg/mL, respectively. The FRAP values of HPI were generally higher than those of HBI and HMI at all tested concentrations, indicating that maturity degree significantly influenced FRAP value of Hawk tea infusions.

**Figure 1:**
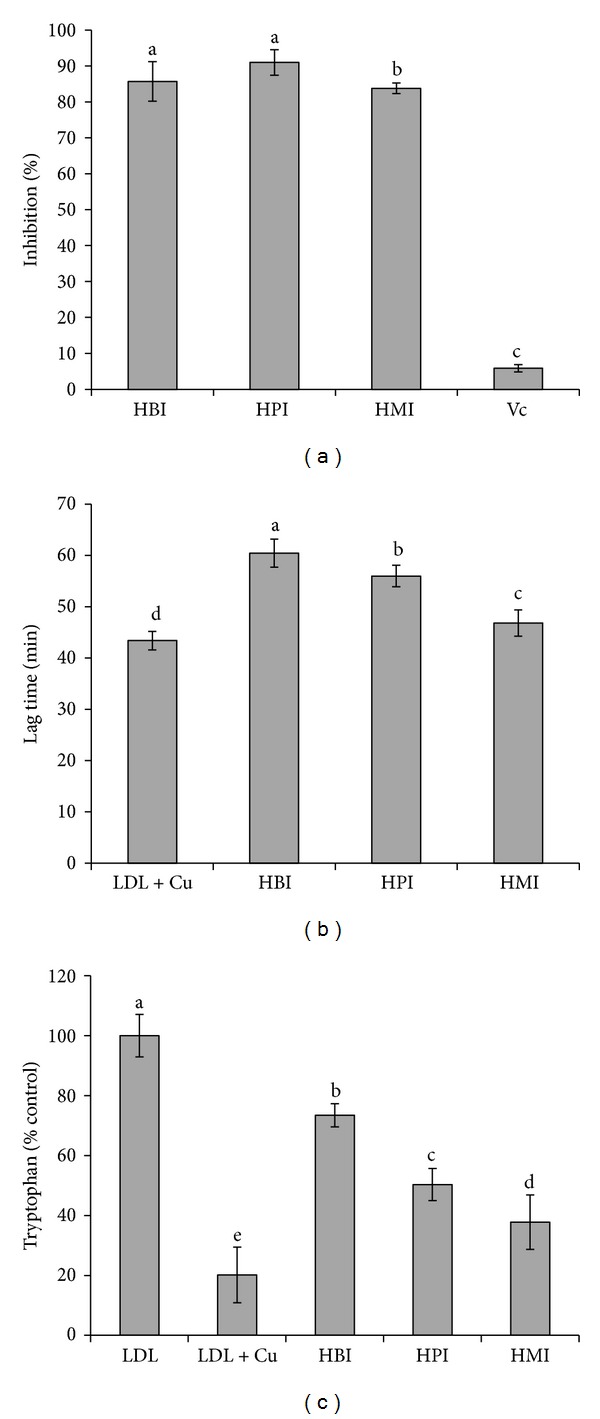
Effects of Hawk tea infusions on hemolysis of human erythrocytes (a); the lag time for formation of conjugated diene (b); and percentage of residual tryptophan fluorescence in LDL incubated in vitro (c). Each value is the mean ± SD; different letters (a, b, c, d, and e) show statistical significance (*P* < 0.05).

*Assay for Erythrocyte Hemolysis*.  Figure 1(a) shows that all samples can inhibit lysis of human erythrocyte. All Hawk tea infusions showed higher ability to inhibit lysis of human erythrocyte than vitamin C (200 mmol/L). The inhibition rate of HMI is 83.80%, even though it is the lowest among the Hawk tea infusions. These results indicate that Hawk tea is an excellent potential candidate for inhibiting lysis of human erythrocyte. Zhu et al. [25] reported that the flavonoid-rich cocoa played a significant role in protection against human erythrocyte hemolysis in adults and exhibited dose-dependent activity.

*Inhibition of LDL Oxidation.* The antioxidants can delay LDL oxidation, which is characterized by the increase of lag time [26]. The lag time can be obtained from monitoring the formation of conjugated diene in the presence of antioxidants. Among the Hawk tea infusions, HBI had the strongest ability to suppress copper-induced LDL oxidation, which prolonged the time to form the conjugated diene by 36% comparing with control, and HMI had the weakest ability, which prolonged the time only by 5% comparing with control (Figure 1(b)). The ability to suppress copper-induced LDL oxidation decreased as the maturity degree of the raw material increased.

Protein oxidation would lead to loss of tryptophan fluorescence, and Figure 1(c) showed the loss of tryptophan fluorescence in LDL. The same with the lag time of LDL to form conjugated diene, all tested samples showed strong ability to inhibit human LDL oxidation, and HBI exhibited stronger ability than HPI and HMI (Figure 1(c)). The results in Figure 1(c) indicate that the activity of suppressing human LDL oxidation decreased with the maturity degree of the raw material increasing.

Lue et al. [27] reported that LDL oxidation was related to the content or species of flavones. HBI showed stronger ability to suppress LDL oxidation than HPI, although it had less content of total flavones than HPI. This result might be due to its high active flavones species or other active constituents.

### 3.3. Correlation between Polyphenol and Antioxidant Activity of Hawk Tea Infusions

Polyphenols are one of the main bioactive components in natural resources and the correlation between polyphenols and their antioxidant activities was discussed in many reports. Zhang et al. [28] reported a positive correlation between the content of total flavonoids and their antioxidant capacity. Wojdyło et al. [29] showed a similar trend between total polyphenols and their antioxidant capacity. Correlation analysis that quantifies the relationship between the antioxidants and antioxidant capacity in Hawk tea infusions was measured by correlation coefficient *r*. The results were shown in Table 4.

The results indicated that DPPH radical scavenging activity was strongly correlated with TPC (*r* = 0.9823) and vitamin C (*r* = 0.9001) in Hawk tea. In all tested constituents, polyphenols were mainly responsible for FRAP. This indicated that polyphenols in plant had powerful capacity to reduce Fe^3+^ to Fe^2+^. In this study, polyphenols in Hawk tea infusions had high affinity with DPPH radical. The species of polyphenols might be different among the three kinds of Hawk teas and further research is needed.

Hackman et al. [30] demonstrated that there was a close relationship between the DPPH radical scavenging activity and polyphenols with low molecular weight in oolong tea. Wojdyło et al. [29] found that the content of total flavonoids was highly positively correlated with DPPH radical scavenging activity in 32 herbs from Poland, which was similar to our results.

Typically, bioactive compounds of plants were produced as secondary metabolites. Primary metabolites, carbohydrates and proteins, were the chemical substances aimed at growth and development. It was reported that peptides [8] and polysaccharide [6] from Hawk tea showed strong antioxidant activity. Therefore, contents of protein and carbohydrate in this work were determined. On the other hand, secondary metabolites, polyphenols and flavonoids, helped plants to increase their overall ability to survive and overcome local challenges by allowing them to interact with their surroundings [31]. It had been reported that bioactive compounds in tea infusion from* Forsythia suspensa* leaves showed a good antioxidant activity [32], and so were tea samples [33]. The antioxidant activity of bioactive compounds might be related to their structure, which had one or more hydroxyl groups and exhibited complex-molecular-mass polymers [34].

## 4. Conclusion

Bioactive components (protein, carbohydrate, vitamin C, total polyphenols, and total flavonoids) and antioxidant activities (DPPH, FRAP, erythrocyte hemolysis, and LDL oxidation) in Hawk tea infusions were investigated. The results indicated that HPI possessed higher content of total polyphenols, almost 79.01 mg/g, but its total flavonoids, carbohydrates, and proteins were much less than HBI. When it comes to antioxidant activity, HPI exhibited the strongest DPPH radical scavenging activity and ferric reducing power. Hence, the polyphenols could be the dominant contributors for HPI's high antioxidant activity.

Our results also indicated that the young leaves of Hawk tea had more contents of all bioactive components than the mature leaves. Furthermore, the young leaves showed higher antioxidant activity than the mature leaves. These results indicated that bioactive components and their antioxidant activity were significantly affected by the maturity degree of the raw material.

## Figures and Tables

**Table 1:** Contents of bioactive constituents in three kinds of Hawk tea infusions.

	Protein (mg/g)	Vc (mg/g)	Carb. (mg/g)	TPC (mg/g)	TFC (mg/g)
HBI	14.36 ± 0.43a	21.67 ± 1.32a	82.79 ± 3.98a	52.79 ± 1.35b	24.65 ± 0.16a
HPI	3.44 ± 0.21c	19.87 ± 0.58a	69.64 ± 7.94b	79.01 ± 1.74a	12.59 ± 0.47b
HMI	8.64 ± 0.41b	3.15 ± 0.11b	35.97 ± 5.56c	4.22 ± 0.55c	1.25 ± 0.02c

Different lowercase letters within the same column are significantly different (*P* < 0.05). Vc: vitamin C; Carb.: carbohydrate; TPC: total polyphenols content; TFC: total flavonoids content.

**Table 2:** DPPH radical scavenging activities of vitamin C, HMI, HPI, and HBI. Each value is the mean ± SD.

DPPH radical scavenging activity (%)					
Con^a^ (mg/mL)	0.1	0.2	0.3	0.4	0.5
HBI	79.51 ± 0.61c	80.55 ± 1.50c	83.01 ± 1.03c	86.00 ± 0.71c	86.15 ± 0.92c
HPI	85.91 ± 0.88b	87.76 ± 0.43b	90.01 ± 0.26b	90.89 ± 0.17b	91.99 ± 0.14b
HMI	54.93 ± 0.61d	61.80 ± 0.43d	66.32 ± 0.16d	69.45 ± 0.30d	75.71 ± 0.98d
Vitamin C	96.38 ± 0.47a	96.47 ± 0.34a	96.50 ± 0.46a	96.83 ± 0.85a	97.00 ± 0.41a

Con^a^: concentration; different letters (a, b, c, and d) show statistical significance (*P* < 0.05).

**Table 3:** FRAP value of HMI, HPI, and HBI. Each value is the mean ± SD.

FRAP value (mmol/L)					
Con^a^ (mg/mL)	0.1	0.2	0.3	0.4	0.5
HBI	0.68 ± 0.03b	1.02 ± 0.11b	1.33 ± 0.02b	1.56 ± 0.01b	1.95 ± 0.03b
HPI	0.97 ± 0.04a	1.50 ± 0.02a	2.20 ± 0.16a	2.45 ± 0.16a	2.60 ± 0.08a
HMI	0.62 ± 0.02b	0.91 ± 0.04b	1.23 ± 0.04c	1.49 ± 0.02c	1.71 ± 0.01c

Con^a^: concentration; different letters (a, b, and c) show statistical significance (*P* < 0.05).

**Table 4:** Correlation matrix of bioactive components in Hawk tea infusions and their antioxidant capacity.

	Protein	Vc	Carbohydrate	TPC	TFC	DPPH	FRAP
Protein	1						
Vc	0.1156	1					
Carbohydrate	0.2986	0.9825	1				
TPC	−0.3196	0.9042	0.8089	1			
TFC	0.5390	0.8990	0.9648	0.6259	1		
DPPH	−0.3286	0.9001	0.8032	0.9823	0.6184	1	
FRAP	−0.6792	0.6505	0.4976	0.9125	0.2521	0.9164	1

Vc: vitamin C; TPC: total polyphenols content; TFC: total flavonoids content.
